# The Impact of Bioabsorbable Nasal Implants, Nasal Radiofrequency Remodeling, and Anesthesia Type on Patient Selection for Nasal Valve Surgery

**DOI:** 10.1002/oto2.70123

**Published:** 2025-05-02

**Authors:** F. Jeffrey Lorenz, Cheng Ma, Scott G. Walen

**Affiliations:** ^1^ Department of Otolaryngology–Head and Neck Surgery Penn State Hershey Medical Center Hershey Pennsylvania USA

**Keywords:** bioabsorbable nasal implant, Latera, lateral wall insufficiency, nasal radiofrequency remodeling, nasal valve collapse, VivAer

## Abstract

**Objective:**

To compare patient demographics, comorbidities, anesthesia type, and trends in nasal valve implantation (NVI) and nasal radiofrequency remodeling (NRR) techniques versus traditional nasal valve repair (NVR).

**Study Design:**

Retrospective case‐control.

**Setting:**

In total, 58 health care organizations (HCOs) across the United States.

**Methods:**

The TriNetX Research Network was queried from 2021 through 2023, forming three cohorts of patients who underwent (1) NVR, (2) NVI, or (3) NRR. Demographics, comorbidities, and anesthesia type were compared across groups at the time of intervention.

**Results:**

A total of 10,568 NVR, 764 NVI, and 485 NRR cases were identified. Patients undergoing NVI or NRR compared to NVR were more likely to be older and exhibit a higher prevalence of medical comorbidities, including sleep apnea, hyperlipidemia, type 2 diabetes, gastroesophageal reflux disease, liver disease, hypertension, ischemic heart disease, other heart diseases, cerebrovascular disease, hearing loss, and kidney disease (all *P* < .05). Of all cases, 82.4% of NVR, 84.8% of NVI, and 55.2% of NRR were performed under general anesthesia. Patients who underwent NRR had the highest comorbidity burden of all cohorts and were most likely to receive local anesthesia. However, when analyzing anesthesia type by specific procedure (NVR, NVI, and NRR), most comorbidities were not significantly more common in those who underwent local anesthesia compared to general anesthesia.

**Conclusion:**

NVI and NRR have provided surgeons with alternative treatment options for nasal valve collapse, especially for patients who are older and with a higher burden of medical comorbidities.

Nasal obstruction is a prevalent condition affecting millions of individuals worldwide, significantly impacting their quality of life.^1^ Among the various anatomic causes of nasal obstruction, dysfunction of the nasal valve area is frequently implicated.^2^ Traditional treatment options for nasal valve dysfunction include a broad spectrum of nasal valve repair (NVR) techniques. These range from minor procedures, such as suture suspension techniques and the removal of excess skin and cartilage,^3^ which can potentially be performed under local anesthesia in an office‐based setting, to more complex surgeries like septorhinoplasty with cartilage grafting (eg, spreader grafts, batten grafts, lateral crural strut grafts, and alar batten grafts, among others) and osteotomies, which involve significant structural changes to the nose and require general anesthesia in the operating room.^4^, ^5^, ^6^

Advancements in medical technology continue to present new minimally invasive therapeutic options, including those that can be administered in the office setting without the need for general anesthesia. Approved by the US Food and Drug Administration (FDA) in 2016, the introduction of the bioabsorbable nasal valve implant (Latera, Stryker) has garnered significant attention as a promising solution for nasal valve dysfunction.^7^ This innovative implant addresses nasal obstruction by providing structural support to the nasal sidewall, thereby enhancing nasal airflow and alleviating symptoms associated with nasal valve collapse. Then, in 2017, nasal radiofrequency remodeling (NRR) (VivAer, Aerin Medical) was introduced, which relies on temperature‐controlled radiofrequency to remodel the lateral nasal wall to prevent collapse.^8^ Both nasal valve implantation (NVI) and NRR have shown subjective and objective improvement in lateral wall insufficiency and symptoms of nasal obstruction, respectively.^9^, ^10^ Advantages of both NVI and NRR are that they are minimally invasive, may be performed easily in the clinic as an outpatient or in the operating room with minimal general anesthetic needs, and have a quicker recovery compared to major nasal surgery.

Now that these treatment options are becoming mainstream, there is a unique opportunity to analyze practice patterns and patient characteristics of those undergoing NVI and NRR. The objective of this study is to determine the demographic and clinical characteristics of patients who undergo NVI and NRR compared to traditional NVR in recent years.

## Methods

The data for this case‐control study were obtained from the TriNetX Research Network (Cambridge, MA). TriNetX is a regularly refreshed database sourced from more than 100 million electronic medical records from over 75 large health care organizations (HCOs) throughout the United States.^11^ The database includes aggregated demographic, diagnosis, procedure, medication, and laboratory data primarily based on diagnosis (International Statistical Classification of Diseases, Tenth Revision [ICD‐10]) and procedure (Current Procedural Terminology [CPT]) codes from inpatient and outpatient medical encounters. TriNetX adheres to the regulations outlined in the Health Insurance Portability and Accountability Act (HIPAA). Given that there was no individually identifiable data, the study (submission ID: STUDY00018629) was exempted from Penn State Institutional Review Board (IRB) review.

The TriNetX Research Network was queried during 2021 through 2023 to identify three different cohorts of patients who underwent treatment for lateral nasal wall insufficiency: (1) controls who underwent traditional surgical repair of nasal vestibular stenosis via any technique (includes minor procedures such as suture techniques to major septorhinoplasty with cartilage grafting) (abbreviated NVR), (2) patients who underwent repair of nasal valve collapse with subcutaneous/submucosal lateral wall implant (ie, Latera) (abbreviated NVI), and finally (3) patients who underwent repair of nasal valve collapse with low energy, temperature‐controlled (ie, radiofrequency) subcutaneous/submucosal remodeling (ie, VivAer, Celon) (abbreviated NRR). Of note, it was not possible to differentiate between (1) specific procedures underwent for NVR or (2) internal and external nasal valve insufficiencies due to limitations regarding diagnosis and procedure codes. As such, the NVR cohort was heterogeneous, and both types of nasal valve insufficiency were included in this study without discrimination. The time frame of interest of 2021 to 2023 was selected because although NVI received FDA approval in 2016, it did not acquire a distinct CPT code for billing until January 1, 2021.^12^ Similarly, although NRR was FDA approved in 2017, it did not obtain a CPT code until January 1, 2023.^13^

The demographic characteristics of each cohort, including age at the time of intervention and sex, were recorded. Race and ethnicity were intentionally not included to prevent bias or stereotypes that could be drawn from comparisons. Clinical characteristics analyzed included major comorbidities across various organ systems diagnosed anytime leading up to or on the day of treatment. These included sleep apnea, hyperlipidemia, type 2 diabetes, gastroesophageal reflux disease (GERD), liver disease, hypertension (HTN), ischemic heart disease, other forms of heart disease (including valve disorders, arrhythmias, and heart failure) cerebrovascular disease, kidney disease, hearing loss, and chronic lung disease. The ICD‐10 and CPT codes utilized to facilitate the analysis are listed in Supplement 1, available online.

Given the potential confounding effect of anesthesia type on outcomes, additional analyses were performed stratifying each cohort by whether the procedure was conducted under general or local anesthesia. Patients were classified as receiving general anesthesia if they had a documented medication code for one of the most commonly used intravenous or inhalational general anesthetics—propofol, sevoflurane, desflurane, or isoflurane—on the date of their nasal valve surgery. Those without documentation of receiving one of these medications were classified as undergoing the procedure with local anesthesia. Lidocaine was excluded as a marker for local anesthesia, as it is commonly utilized in the setting of both local and general anesthesia. Demographics and comorbidities were compared for each procedure based on anesthesia type.

As an additional analysis, procedural volume by year was analyzed for NVR and NVI. This was not possible for NRR as it only obtained a unique CPT code in 2023, so only 1 year of data were available for analysis.

All statistical analyses were performed within the TriNetX platform, which utilizes Java, R, and Python programming software. The mean ages for each cohort were compared utilizing *t* test difference in means. The remainder of the demographics and comorbidities were compared utilizing odds ratios and associated *P* values. Statistical significance was defined as *P* < .05.

## Results

The total cohort included 10,568 patients who underwent NVR, 764 who underwent NVI, and 485 who underwent NRR during the study period. The demographics and comorbidities of each group are presented in Table 1. On average, patients in the NVI and NRR cohorts were significantly older at the time of intervention compared to patients who underwent NVR. There were more males in both the NVI and NRR cohorts compared to the NVR cohort, though this was only statistically significant for NRR.

**Table 1:** Demographic and Clinical Characteristic Comparisons of Patients Who Underwent Surgical Nasal Valve Repair (n = 10,568), Nasal Valve Implants (n = 764), and Nasal Radiofrequency Remodeling (n = 485)^a^

Demographic	Surgical nasal valve repair (n = 10,568)	Nasal valve implant (n = 764)	OR (95% CI)	*P* value	Nasal radiofrequency remodeling (n = 485)	OR (95% CI)	*P* value
Age, y	41.1 ± 17.1	48.8 ± 16.4	N/A	<.001	52.6 ± 16.6	N/A	<.001
Sex							
Male	5165 (49%)	399 (52%)	1.14 (0.99‐1.32)	.07	267 (55%)	1.28 (1.07‐1.54)	.008
Female	5317 (50%)	325 (43%)	0.73 (0.63‐0.85)	<.001	216 (45%)	0.79 (0.66‐0.95)	**.01**
Unknown	86 (1%)	40 (5%)	6.73 (4.59‐9.87)	<.001	2 (<1%)	0.50 (0.12‐2.06)	.34
Comorbidities							
Sleep apnea	2188 (21%)	234 (31%)	1.69 (1.44‐1.99)	<.001	207 (43%)	2.85 (2.37‐3.43)	<.001
Hyperlipidemia	2277 (22%)	275 (36%)	2.05 (1.75‐2.39)	**<.001**	237 (49%)	3.48 (2.89‐4.18)	**<.001**
Type 2 diabetes	651 (6%)	72 (9%)	1.59 (1.23‐2.05)	<.001	86 (18%)	3.28 (2.57‐4.20)	<.001
GERD	2588 (24%)	236 (31%)	1.38 (1.17‐1.62)	<.001	210 (43%)	2.35 (1.96‐2.83)	**<.001**
Liver disease	576 (5%)	59 (8%)	1.45 (1.10‐1.92)	.009	64 (13%)	2.64 (2.00‐3.48)	<.001
HTN	2273 (21%)	240 (31%)	1.67 (1.42‐1.96)	<.001	226 (47%)	3.18 (2.65‐3.83)	**<.001**
Ischemic heart disease	530 (5%)	68 (9%)	1.85 (1.42‐2.41)	**<.001**	67 (14%)	3.04 (2.31‐3.99)	**<.001**
Other forms of heart disease	1291 (12%)	128 (17%)	1.45 (1.19‐1.76)	<.001	134 (28%)	2.74 (2.23‐3.38)	**<.001**
Cerebrovascular disease	298 (3%)	37 (5%)	1.75 (1.24‐2.49)	**.002**	40 (8%)	3.10 (2.20‐4.37)	<.001
Kidney disease	381 (4%)	48 (6%)	1.79 (1.32‐2.44)	**<.001**	51 (11%)	3.14 (2.31‐4.27)	**<.001**
Hearing loss	647 (6%)	117 (15%)	2.77 (2.24‐3.43)	**<.001**	82 (17%)	3.12 (2.43‐4.01)	<.001
Chronic lung disease	2097 (20%)	171 (22%)	1.16 (0.98‐1.39)	.09	149 (31%)	1.79 (1.47‐2.19)	<.001

Abbreviations: CI, confidence interval; GERD, gastroesophageal reflux disease; HTN, hypertension; OR, odds ratio.

^a^ Odds ratios and 95% CIs compare patients who underwent nasal valve implants or nasal radiofrequency remodeling to controls who underwent surgical nasal valve repair.

Regarding comorbidities, compared to patients who underwent NVR, patients who underwent NVI were significantly more likely to have an existing diagnosis of sleep apnea, hyperlipidemia, type 2 diabetes, GERD, liver disease, HTN, ischemic heart disease, other forms of heart disease, cerebrovascular disease, kidney disease, and hearing loss. There were no significant differences in chronic lung disease between these two groups (Table 1). Patients who underwent NRR were significantly more likely to carry a diagnosis of all analyzed comorbidities at the time of intervention compared to NVR. Patients who underwent NRR had the greatest rates of all comorbidities compared to those who underwent NVI (Table 1).

Analyses stratified by whether specific procedural interventions were performed under general or local anesthesia are presented in Table 2. There were 82.4% of NVR cases, 84.8% of NVI cases, and 55.2% of NRR cases performed under general anesthesia, with the remainder performed under local anesthesia. For NVR, there were no differences in age, sex, or rates of type 2 diabetes, liver disease, ischemic heart disease, cerebrovascular disease, or hearing loss in patients who underwent general versus local anesthesia. Patients who underwent general anesthesia were more likely to have a coexisting diagnosis of sleep apnea, hyperlipidemia, GERD, HTN, other forms of heart disease, kidney disease, and chronic lung disease. Patients who underwent NVR under local anesthesia did not have any increased rates of comorbidities compared to those who underwent NVR under general anesthesia. Patients who underwent NVI under local anesthesia were significantly older and more likely to have a coexisting diagnosis of sleep apnea compared to those who underwent NVI under general anesthesia. There were no other significant differences in demographics or comorbidities. Finally, patients who underwent NRR under local anesthesia were more likely to be older and have a diagnosis of hearing loss, and less likely to have a diagnosis of HTN or chronic lung disease compared to those who underwent NRR under general anesthesia. There were no significant differences in other demographics or comorbidities.

**Table 2:** Demographic and Clinical Characteristic Comparisons of Patients Who Underwent Surgical Nasal Valve Repair (n = 10,568), Nasal Valve Implants (n = 764), and Nasal Radiofrequency Remodeling (n = 485) Under General Versus Local Anesthesia

Demographic	Surgical nasal valve repair general anesthesia (n = 8706)	Surgical nasal valve repair local anesthesia (n = 1862)	OR (95% CI)	*P* value	Nasal valve implant general anesthesia (n = 648)	Nasal valve implant local anesthesia (n = 116)	OR (95% CI)	*P* value	Nasal radiofrequency remodeling general anesthesia (n = 268)	Nasal radiofrequency remodeling local anesthesia (n = 217)	OR (95% CI)	*P* value
Age, y	41.2 ± 17	40.5 ± 17.5	N/A	.12	48.3 ± 16.3	51.8 ± 16.9	N/A	**.03**	48.5 ± 16.1	57.7 ± 15.8	N/A	**<.001**
Sex												
Male	4256 (49%)	909 (49%)	1.00 (0.90‐1.10)	.96	341 (53%)	58 (50%)	0.90 (0.61‐1.34)	.60	149 (56%)	118 (44%)	0.95 (0.66‐1.36)	.79
Female	4369 (50%)	948 (51%)	1.03 (0.93‐1.14)	.57	271 (42%)	54 (47%)	1.21 (0.81‐1.80)	.34	118 (44%)	98 (45%)	1.05 (0.73‐1.50)	.80
Unknown	81 (1%)	5 (<1%)	0.29 (0.12‐0.71)	.007	36 (6%)	4 (3%)	0.61 (0.21‐1.74)	.35	1 (<1%)	1 (<1%)	1.24 (0.08‐19.88)	.88
Comorbidities												
Sleep apnea	1888 (22%)	300 (16%)	0.69 (0.61‐0.79)	**<.001**	188 (29%)	46 (40%)	1.61 (1.07‐2.42)	**.02**	107 (40%)	100 (46%)	1.29 (0.90‐1.85)	.17
Hyperlipidemia	1921 (22%)	356 (19%)	0.83 (0.74‐0.95)	.005	229 (35%)	46 (40%)	1.20 (0.80‐1.80)	.37	133 (50%)	104 (48%)	0.93 (0.65‐1.34)	.71
Type 2 diabetes	548 (6%)	103 (6%)	0.87 (0.70‐1.08)	.21	60 (9%)	12 (10%	1.13 (0.59‐2.17)	.71	48 (18%)	38 (18%)	0.97 (0.61‐1.56)	.91
GERD	2235 (26%)	353 (19%)	0.68 (0.60‐0.77)	<.001	198 (31%)	38 (33%)	1.11 (0.73‐1.69)	.64	124 (46%)	86 (40%)	0.76 (0.53‐1.10)	.14
Liver disease	485 (6%)	91 (5%)	0.87 (0.69‐1.10)	.24	48 (7%)	11 (9%)	1.31 (0.66‐2.60)	.44	36 (13%)	28 (13%)	0.95 (0.56‐1.62)	.86
HTN	1917 (22%)	356 (19%)	0.84 (0.74‐0.95)	**.006**	203 (31%)	37 (32%)	1.03 (0.67‐1.57)	.90	138 (51%)	88 (41%)	0.64 (0.45‐0.92)	.02
Ischemic heart disease	449 (5%)	81 (4%)	0.84 (0.66‐1.07)	.15	55 (8%)	13 (11%)	1.36 (0.72‐2.58)	.35	35 (13%)	32 (15%)	1.15 (0.69‐1.93)	.59
Other forms of heart disease	1093 (13%)	198 (11%)	0.83 (0.71‐0.97)	**.02**	103 (16%)	25 (22%)	1.45 (0.89‐2.37)	.13	76 (28%)	58 (27%)	0.92 (0.62‐1.38)	.69
Cerebrovascular disease	242 (3%)	56 (3%)	1.08 (0.81‐1.46)	.59	27 (4%)	10 (9%)	2.17 (1.02‐4.61)	.04	20 (7%)	20 (9%)	1.26 (0.66‐2.41)	.49
Kidney disease	331 (4%)	50 (3%)	0.70 (0.52‐0.94)	**.02**	38 (6%)	10 (9%)	1.51 (0.73‐3.13)	.26	22 (8%)	29 (13%)	1.72 (0.96‐3.10)	.07
Hearing loss	545 (6%)	102 (5%)	0.87 (0.70‐1.08)	.20	101 (16%)	16 (14%)	0.87 (0.49‐1.53)	.62	35 (13%)	47 (22%)	1.84 (1.14‐2.98)	.01
Chronic lung disease	1820 (21%)	277 (15%)	0.66 (0.58‐0.76)	<.001	139 (21%)	32 (28%)	1.40 (0.89‐2.18)	.15	93 (35%)	56 (26%)	0.65 (0.44‐0.97)	.04

Abbreviations: CI, confidence interval; GERD, gastroesophageal reflux disease; HTN, hypertension; OR, odds ratio.

Analysis of procedural volume by year for NVR, NVI, and NRR is demonstrated in Table 3. Across 2021 to 2023, the procedural volume and HCOs performing NVR and NVI remained stable. All cases of NRR were documented in 2023, as this was the first year it was allocated a unique CPT code.

**Table 3:** Yearly Case Volume of Surgical Nasal Valve Repair (n = 10,568), Nasal Valve Implants (n = 764), and Nasal Radiofrequency Remodeling (n = 485) During 2021 to 2023^a^

	2021 case volume	2021 HCOs	2022 case volume (% change)	2022 HCOs (% change)	2023 case volume (% change)	2023 HCOs (% change)
Surgical nasal valve repair	3648	56	3513 (−3.7%)	55 (−1.8%)	3407 (−3.0%)	56 (+1.8%)
Nasal valve implant	254	33	254 (0%)	35 (+6.1%)	256 (+0.7%)	32 (−8.6%)
Nasal radiofrequency remodeling	‐	‐	‐	‐	485	25

Abbreviation: HCO, health care organization.

^a^ % change compares the given year to the year prior.

## Discussion

For elderly patients or those burdened with comorbidities, the heightened risk of prolonged general anesthesia may render elective nasal surgery unjustifiable. Conversely, noninvasive options like nasal cones or strips, while offering temporary relief and avoiding surgical risks,^14^ are often limited by their nighttime wear requirement and patient intolerance due to discomfort, inconvenience, skin reactions, or visibility concerns. Fortunately, minimally invasive procedural treatments for nasal valve collapse have emerged, offering significant benefits with minimal anesthetic risks. Importantly, research has supported the effectiveness of both NVI and NRR. One prospective study by Sidle et al demonstrated a clinically significant improvement in nasal obstruction symptom evaluation (NOSE) scores (≥30 points) in 73% of patients who underwent NVI and 83% who underwent NVI and inferior turbinate reduction.^9^ Furthermore, another prospective study by Jacobowitz et al demonstrated an average reduction in NOSE scores from 81.0 to 21.6 at 6 months following NRR, which persisted throughout the entire 4‐year follow‐up period.^10^

In the current study, patients opting for NVI or NRR were more likely to be older males with higher comorbidity rates compared to those undergoing traditional NVR. Notably, conditions such as sleep apnea, hyperlipidemia, type 2 diabetes, cardiovascular diseases, and others were more prevalent in the NVI and NRR cohorts. These findings suggest that otolaryngologists are recommending these minimally invasive interventions to patients with underlying systemic conditions, given their shorter recovery times and reduced perioperative risks compared to major NVR surgeries like open septorhinoplasty. This may also imply that individuals previously excluded from surgical intervention due to anesthesia‐related risks can now access treatment through NVI or NRR, highlighting the growing accessibility of these procedures for previously overlooked populations.

Given the elective nature of nasal surgery for nasal obstruction, appropriate patient selection is essential. Traditionally, surgical candidates for septoplasty or rhinoplasty skewed towards younger individuals with fewer comorbidities and a higher tolerance for anesthesia, indicative of a longer life expectancy.^15^ In one study of patients undergoing rhinoplasty, age older than 40 years was an independent risk factor for major surgical or anesthetic complications, such as hematoma, infection, pulmonary issues, and deep vein thrombosis/pulmonary embolism.^16^ Another study reported that patients with previous myocardial infarction, a history of HTN, and renal failure face increased risk of cardiovascular death following elective surgery under general anesthesia.^17^ As the American Society of Anesthesiologists Physical Status Classification (ASA Class) rises, signifying a worsening preoperative health status, the probability of medical complications and mortality linked to any surgical procedure increases exponentially.^18^ In contrast, the complications of NVI and NRR are confined to local adverse reactions such as abscess and device extrusion reported for Latera,^19^ and extremely rare complications like synechiae formation, mucosal perforation, and empty nose syndrome for VivAer.^20^, ^21^ These are relatively innocent compared to complications of prolonged general anesthesia. In the current study, patients who underwent NRR had the highest rates of comorbidities, and this was also the procedure most likely to be performed under local anesthesia. Even if performed in an operating room, as was the case for 4/5 of NVI and half of NRR procedures, the reduced duration of general anesthesia significantly diminishes associated risks. Notably, these less invasive options still offer significant improvement in nasal obstruction and the integrity of the lateral nasal wall while minimizing perioperative risks.^9^, ^21^

Interestingly, although both NVI and NRR patients had increased comorbidities compared to NVR, patients who underwent NRR had the greatest odds for all comorbidities. For example, patients who underwent NVI had 1.67 times increased odds of having a preexisting diagnosis of HTN compared to those who underwent NVR, whereas those who underwent NRR had 3.18 times the odds of HTN. The reason for this is not entirely clear from the data; however, it is possible that some patients treated with NVI also underwent concomitant septoplasty or inferior turbinate reduction, necessitating general anesthesia and prolonged operating room time. Some otolaryngologists may have favored VivAer for patients with greater comorbidities due to its versatility in addressing conditions beyond nasal valve collapse, such as septal swell body and inferior turbinate hypertrophy, and its avoidance of introducing foreign bodies.

It is unclear why, when comparing the demographics and comorbidities of patients undergoing NVR, NVI, and NRR procedures under local versus general anesthesia, the presence of comorbidities did not significantly influence the likelihood of receiving general or local anesthesia. One possible explanation is that both patient and physician preferences may have played a role in the decision to perform the procedure under general anesthesia. Surgeons may have opted for general anesthesia based on comfort, experience, or the belief that even patients with comorbidities can tolerate short procedures safely under general anesthesia. Additionally, patients who prefer the comfort of general anesthesia may have influenced the decision, regardless of their comorbidity profile.

One potential hypothesis for the differences in sex in treatment choices—more females opting for NVR while males lean towards NVI or NRR—could stem from differing perceptions of functional concerns and nasal esthetics between males and females. It is plausible that males, who may prioritize functional improvement over cosmetic considerations, are more inclined towards minimally invasive options like NVI and NRR to address nasal obstruction. Conversely, females, who may place greater emphasis on cosmetic outcomes, might opt for traditional NVR, which has the potential to offer more extensive surgical correction of both functional and esthetic issues. Furthermore, older males may undergo NVI or NRR with higher frequency due to the unique valve stenosis that arises from thick skin, ligament and cartilage weakness, and tip ptosis common in this group. This is often the same impetus for performing the rhinolift procedure for aging patients, which involves excision of redundant supratip skin and suspension of the lower lateral cartilages to the upper lateral cartilages to address nasal valve insufficiency.^22^, ^23^ The effectiveness of NVI and NRR in treating nasal obstruction in the aging nose highlights an area for future research.

A previous study, conducted before the introduction of a specific CPT code for NVI, indicated a notable increase in nasal vestibular repair volume in 2017, coinciding with the FDA approval of NVI. This surge was accompanied by a rise in the number of unique providers performing nasal vestibular repair.^24^ In our analysis of 2021 through 2023, it seems that NVI volume has reached a plateau in the number of annual procedures and HCOs performing them. This stabilization could be attributed to the availability of multiple treatment options for nasal valve collapse. Only 1 year of data were available for the NRR analysis; therefore, further investigation is warranted to analyze NRR trends over the coming years as more data emerges.

The strengths of this study lie in its utilization of a large, multicenter database, allowing for comprehensive analysis of patients undergoing NVI, NRR, and NVR across diverse patient populations and HCOs. Additionally, this represents the first database study since the implementation of unique CPT codes for NVI and NRR. However, several limitations warrant consideration. First, the group described as NVR in this study is not a uniform group since it likely included a range of patients undergoing both minor (small suture and grafting techniques) and major (functional septorhinoplasty) procedures. In this study, there was no way to determine the specific type of NVR intervention patients underwent as all were classified under the same CPT code. Although we explored additional analyses filtering by anesthesia type, we were unable to filter based on time under anesthesia, which is another important component of anesthetic risk taken into account by surgeons when deciding on optimal procedural interventions. Although we were able to determine the rates of numerous comorbidities, the absence of patient‐level data hinders insight into the individualized decision‐making process behind opting for less invasive interventions. Additionally, relying on ICD and CPT codes for patient selection may introduce inaccuracies and incomplete data capture. There was likely a large volume of procedures performed outside of the HCOs included in this database, for example, in private practice settings. The indications for NRR are not solely limited to nasal valve insufficiency; therefore, it is possible that some patients included in the cohort were treated for isolated inferior turbinate hypertrophy. Lastly, this study solely outlines the demographic and clinical characteristics of patients undergoing these procedures and does not evaluate their efficacy. It is essential to note that candidates for NVI or NRR are not exclusively older or burdened with comorbidities; younger, healthier individuals may also opt for these interventions due to preference or surgery‐related apprehensions, for example.

## Conclusions

Patients undergoing NVI or NRR are more likely to be older and present with a higher prevalence of comorbidities compared to those opting for traditional NVR. The adoption of minimally invasive interventions such as NVI and NRR has expanded accessibility to new patient populations, particularly those previously deemed unsuitable for traditional surgical repair due to age or anesthetic risks.

## Author Contributions

**F. Jeffrey Lorenz**, concept design, data collection, reviewing data analysis, writing manuscript; **Cheng Ma**, concept design, reviewing data analyses, critical editing of manuscript and final approval; **Scott G. Walen**, concept design, reviewing data analyses, critical editing of manuscript and final approval.

## Disclosures

### Competing interests

The authors declare that there is no conflict of interest.

### Funding source

The project was supported by the National Center for Advancing Translational Sciences, National Institutes of Health (NIH), through Grant UL1 TR002014.

## Supporting information


**Supplement 1:** Diagnosis (ICD‐10) and Procedure (CPT) Codes
The diagnosis and procedure codes utilized to execute the database query are listed.
