# Supplementary material for: The Impact of Bioabsorbable Nasal Implants, Nasal Radiofrequency Remodeling, and Anesthesia Type on Patient Selection for Nasal Valve Surgery
Source: OTO Open. 2025 May 2;9(2):e70123. doi: 10.1002/oto2.70123 (PMC12046381; doi:10.1002/oto2.70123)
Supplement: Supplementary file 1 — Supplement 1: Diagnosis (ICD‐10) and Procedure (CPT) Codes The diagnosis and procedure codes utilized to execute the database query are listed. [file OTO2-9-e70123-s001.docx]

**Supplement 1: Diagnosis (ICD-10) and Procedure (CPT) Codes**

**Procedures:**

- Surgical repair for nasal vestibular stenosis (NVR, CPT 30465)
- Repair of nasal valve collapse with subcutaneous/submucosal lateral wall implant(s) (NVI, CPT 30468)
- Repair of nasal valve collapse with low energy, temperature-controlled (radiofrequency) subcutaneous/submucosal remodeling (NRR, CPT 30469).

**Diagnoses:**

- Sleep apnea G47.3
- Hyperlipidemia E78
- Type 2 diabetes E11
- Gastroesophageal reflux disease (GERD) K21
- Liver disease K7-K77
- Hypertension (HTN) I10-I1A
- Ischemic heart diseases I20-I25
- Other forms of heart diseases (including valve disorders, arrhythmias, and heart failure) I30-I52
- Cerebrovascular diseases I30-I5A
- Kidney diseases N17-N19
- Hearing loss H90
- Chronic lung diseases J40-J4A

**Medications:**

- Propofol 8782
- Sevoflurane 36453
- Desflurane 27340
- Isoflurane 6026
